# First-Principles Investigation to Ionization of Argon Under Conditions Close to Typical Sonoluminescence Experiments

**DOI:** 10.1038/srep20623

**Published:** 2016-02-08

**Authors:** Wei Kang, Shijun Zhao, Shen Zhang, Ping Zhang, Q. F. Chen, Xian-Tu He

**Affiliations:** 1 HEDPS, Center for Applied Physics and Technology, Peking University, Beijing 100871, China; 2 College of Engineering, Peking University, Beijing 100871, China; 3 Institute of Applied Physics and Computational Mathematics, Beijing 100088, China; 4 National Key Laboratory of Shock Wave and Detonation Physics, Institute of Fluid Physics, P. O. Box 919-102, Mianyang, Sichuan, China

## Abstract

Mott effect, featured by a sharp increase of ionization, is one of the unique properties of partially ionized plasmas, and thus of great interest to astrophysics and inertial confinement fusion. Recent experiments of single bubble sonoluminescence (SBSL) revealed that strong ionization took place at a density two orders lower than usual theoretical expectation. We show from the perspective of electronic structures that the strong ionization is unlikely the result of Mott effect in a pure argon plasma. Instead, first-principles calculations suggest that other ion species from aqueous environments can energetically fit in the gap between the continuum and the top of occupied states of argon, making the Mott effect possible. These results would help to clarify the relationship between SBSL and Mott effect, and further to gain an better understanding of partially ionized plasmas.

A sharp rising in ionization is a remarkable feature of Mott effect^1^ in partially degenerate plasmas. The effect is also known as Mott transition^2^, pressure ionization^3^, or plasma phase transition (PPT)^4^ on various occasions. It indicates the disintegration of bounded states and the formation of extended states with reduced ionization potential (IP). Usually, a companion structural transition is also expected together with the steep change of ionization^5^. It is arguably the most prominent nonideality character of partially ionized plasmas of strong coupling^2^, a focus of current studies of warm dense matter (WDM). Mott effect is therefore of particular interest to astrophysics^6^,^7^ as well as inertial confinement fusion^8^,^9^.

The physical origin of Mott effect, however, has been under debate since it was first noticed in 1940’s^10^. It is considered an result either derived from the competition between geometric confinement and the spatial extension of wave functions, i.e., the picture of pressure ionization^2^,^3^, or from a balance between screened Coulomb interaction and quantum repulsion, i.e., the picture of PPT^4^,^11^. These two pictures are closely related but have subtle differences. Nevertheless, both theories suggest a transition taking place at such density that atomic distance is comparable with the diameter of the outer-most electronic orbital. This prediction was well supported by recent experiments and first-principles calculations on dense hydrogen systems^12^,^13^,^14^,^15^,^16^, where a phase transition with abrupt increasing in ionization was observed around *ρ* ~ 1.0 g/cm^3^, in line with the theoretical expectation.

However, in single bubble sonoluminescence (SBSL) experiments^17^,^18^,^19^, where noble gas, e.g., argon (Ar), was compressed by the collapse of the cavity in aqueous solutions^20^, a steep increase of ionization was observed at a number density *ρ*_*N*_ ~ 10^21^/cm^3^, i.e., ~0.07 g/cm^3^, and at a temperature *T* ~ 1 eV, i.e., 11000 K. The *ρ*_*N*_ corresponds to an average atomic distance of 10 Å, which is much larger than the length scale (~2 Å) of the outer-most 3*p* electronic orbital of Ar atoms. A theory developed recently by Kappus *et al.*^21^ suggested that sonoluminescence was essentially the result of Mott effect in noble-gas-element plasmas. They proposed a classical chemical model with Debye-Hückle screening, which showed that the lowering of IP was greatly enhanced by the ionization process, making a sharp increase in ionization possible at a much lower density than that predicted by prior theories^22^. If so, this would lead to a significant revision to the physical picture of Mott effect, and consequently an updated understanding of the ionization mechanism of partially degenerate plasmas.

However, as was pointed out by Redmer and Holst^1^, the appearance of Mott effect in a theory is sensitively relied on the details of the effective interaction at the distance comparable with the size of an atom. It would be desirable if a method can take as much as possible the details of the interaction into consideration. Following Knudson *et al.*^16^, Morales *et al.*^12^, Lorenzen *et al.*^23^, and Scandolo^24^, one can alternatively use first-principles method to detect Mott effect. Their method probes the change in pressure induced by the companion structural transition. The method works well as long as its resolution overcomes the finite-size effect^12^,^23^,^24^, which, as they revealed, could be attained in a calculation composed of 400–500 atoms. However, it should be noted that the method itself is difficult to exclude the possibility of a transition beyond its resolution. Under this condition, information provided by other properties is then critical to pin down the problem. Electronic structures are a suitable candidate to serve this purpose. They are expected to go through a remarkable change in order to accommodate the sharp increase of ionization when Mott effect takes place. As a consequence, the effective IP will be greatly reduced, which can be seen through a simple estimation with Saha’s equation^3^. More importantly, electronic structures are less constrained by the finite-size effect. They are locally determined near the chemical potential (or Fermi level at zero temperature) in an amorphous system because of the randomness of ionic positions. This localization was first illustrated by Anderson^25^ and was observed in electronic structure calculations on a variety of WDM systems^26^,^27^,^28^. It greatly reduces the system size in a calculation from over 400 atoms to ~40 atoms without notable changes in electronic structures.

In this work, we concentrate on electronic structures of Ar at equilibrium, calculated using the first-principles molecular dynamics (FPMD) method. In the calculation, ions are described via classical Newtonian molecular dynamics (MD), whereas electrons are treated quantum-mechanically through density functional theory (DFT). Two levels of quantum descriptions are used in the calculation. Mean-field effects of excited electrons are accounted for through finite-temperature DFT (FTDFT) formula proposed by Mermin^29^, which assigns the population of excited electrons according to the Fermi-Dirac distribution. Dynamical effects associated with electronic excitations^30^, are taken into consideration through real-time time-dependent DFT (TDDFT) method^31^, which explicitly describes the evolution of electronic wave functions with reasonable accuracy^32^,^33^. In both methods, local density approximation (LDA)^34^ is used to describe the exchange-correlation interaction between electrons. These two FPMD methods are referred as FTDFT+MD and TDDFT+MD respectively hereinafter. A detailed account can be found in the methods section.

The inaccuracy brought about by the methodology is also considered. It has been well known that LDA underestimates the energy gap between occupied and unoccupied states at low temperature, usually 40–60% for semiconductors and insulators^27^,^35^. Electronic structures thus calculated favor the occurrence of Mott effect as a result of severe overestimation to the ionization. We show that even taking the overestimation into account, the sharp increase of ionization in SBSL experiments is unlikely the result of Mott effect in a pure Ar plasma system. Instead, ions and cations originated from dissociated water molecules are energetically located in the energy gap. They possibly provide the necessary energy levels to essentially reduce the effective IP, which can further trigger the Mott effect.

## Results and Discussion

### Thermodynamical Properties

In a typical SBSL experiment containing Ar, a sharp increase of ionization takes place with strong visible light emitted at a pressure over 4000 bar and at a temperature ~1.5 eV (17000 K)^20^,^36^. This temperature is far beyond the liquid-gas critical temperature ~150 K^37^. The number density of Ar ion *n*_*i*_ is approximately 4 × 10^21^/cm^3^, i.e., 0.25 g/cm^3^, about half of the number density at the liquid-gas critical point. However, surprisingly, the number density of electrons *n*_*e*_ is roughly the same as or even higher than *n*_*i*_, making the degree of ionization *α* ≥ 1. The coupling constant^2^ Γ is then greater than 2, which indicates the Ar plasma is strongly coupled.

Testing calculation on experimental conditions shows that electronic structures and IP are essentially the same as those of an isolated Ar atom. The interaction between Ar atoms is thus negligible according to the quantum perturbation theory. Compared with experimental value of 15.7 eV, the calculation (10.3 eV) underestimates the IP by 33%. *α* is therefore overestimated by about 5 times, as estimated using *α* ~ exp(−*I*_0_/2*T*)^38^ with *I*_0_ the magnitude of IP. Even with this overestimation, the calculated *α* is less than 3%, which is about twice the value of 1.4 % given by the self-consistent fluid variational theory(SCFVT)^39^ and essentially implies no Mott effect.

However, the absence of Mott effect is possibly caused by the drift of temperature and density predicted by the calculation, which is inevitable for the FTDFT+MD method due to various approximations involved in the calculation, as has been noticed in the calculation of hydrogen^12^,^16^. A detailed discussion on the influence of the approximations, including the semi-local approximation to the exchange-correlation functional and the neglecting of nuclear quantum effects, is carefully presented in the recent work of Knudson *et al.*^16^. To further exclude this possibility, a series of FTDFT+MD calculations are carried out surrounding the experimental density and temperature. Based on previous theoretical estimations^4^,^11^,^22^, which predicted that Mott effect took place at a length scale comparable to the diameter of the outer-most electronic orbitals, we consider the density between 1 g/cm^3^ and 8.34 g/cm^3^. The upper limit of 8.34 g/cm^3^ corresponds to an average atomic distance ~2 Å, slightly larger than the diameter *d* = 1.94 Å of the 3*p* orbital for an isolated Ar atom. The diameter of the 3*p* orbital is calculated as 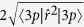, where 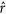 is the radius operator, and the wave function 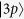 is extracted from the all electron atomic DFT-LDA calculation of Ar atom. This definition follows Atzeni and Meyer-ter-Vehn in the discussion of the lowering of ionization potential^40^. The lower bound corresponds to the density at which interactions between atoms start to make observable changes in their electronic structures. The temperature concerned is between 10^3^ K and 2 × 10^4^ K. Higher temperature is not considered because both theories and experiments showed that Mott effect prefers lower temperature. It can only take place below some critical temperature^4^,^11^,^12^,^13^,^21^,^22^.

**Figure 1:**
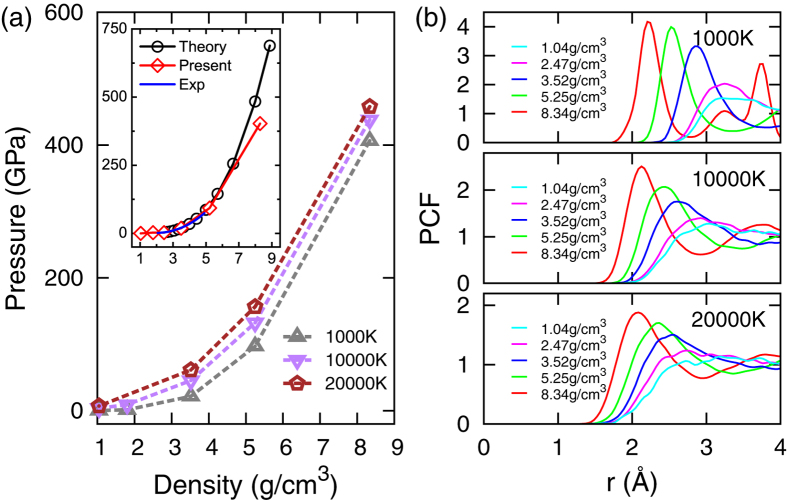
(**a**) Pressure isotherms of Ar calculated using the FTDFT+MD method at T = 10^3^ K, 10^4^ K, and 2 × 10^4^ K. Displayed in the inset is the pressure isotherm at room temperature, compared with experimental measurements of Shimizu *et al.*^41^, and Monte Carlo calculation of Ross *et al.*^42^ with an empirical pair interaction. (**b**) Pair correlation function *g*(*r*) along the isotherms displayed in (**a**), which indicates short-range structures of Ar plasma.

Figure 1(a) displays pressure isotherms of Ar calculated using the FTDFT+MD method at T = 10^3^ K, 10^4^ K, and 2 × 10^4^ K, where isotherms of T = 10^3^ K and T = 2 × 10^4^ K define the boundary of our calculations, and the isotherm of T = 10^4^ K indicates the median of the temperature measured in SBSL experiments. To give an estimation to the reliability of the FTDFT+MD method, also displayed in the inset is the calculated isotherm at room temperature, compared with experimental results measured by Brillouin scattering^41^ and theoretical results calculated using Monte Carlo method together with an empirical pair interaction^42^. The calculated pressure agrees well with the experimental result, but deviates from the Monte Carlo calculation at densities higher than 8 g/cm^3^. This deviation is expected because the atomic distance at that density is only slightly larger than the diameter (1.94 Å) of the 3*p* orbital of Ar atom, where tunneling effect cannot be well accounted for by the empirical pair potential fitted from experimental data at low densities. In Fig. 1(b), pair correlation functions *g*(*r*) along the isotherms are presented to reveal structural changes. When a first-order phase transition takes place, *g*(*r*) usually undergoes a significant change. This, however, is only observed at *ρ* = 8.34 g/cm^3^ and T = 10^3^ K in Fig. 1(b), which is an extreme high density and low temperature condition compared with that in SBSL experiments^20^,^36^. Except that case, *g*(*r*) and isotherms in Fig. 1 display a smooth change with respect to *T* and *ρ*. It shows that structural transition is not a prominent feature near the experimental conditions, which is consistent with the usual understanding of Mott effect^2^,^4^,^5^, and is supported by FTDFT+MD calculations on warm dense helium^43^.

### Electronic Structures

In addition to thermodynamical properties, the electronic structure of Ar plasma is also calculated using the FTDFT+MD method. A strong reduction (over 75 %) in IP is expected according to the estimation of Kappus *et al.*^44^ on experimental results of Xe. The IP, estimated as the energy gap across the chemical potential in the calculation, can then be used as an indication to the occurrence of Mott effect. In the FTDFT+MD method, the IP lowering is assessed with the help of Ewald summation technique^34^, and the screened interaction between Ar ions is calculated using DFT through Hellmann-Feynman theorem^34^ or Ehrenfest theorem^30^. These treatments are more sophisticated than the Debye-Hückle screening model and the IP lowering model (i.e., 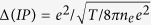). In particular, it self-consistently includes the enhancement of ionization to the lowering of IP. Calculations on hydrogen systems^12^,^16^,^23^,^24^ have shown that they are capable to predict the existence of Mott effect. However the calculated transition point sensitively depends on the details of the calculation, e.g., the choice of density functional and the inclusion of nuclear quantum effect, as has been carefully discussed by Knudson *et al.*^16^.

**Figure 2:**
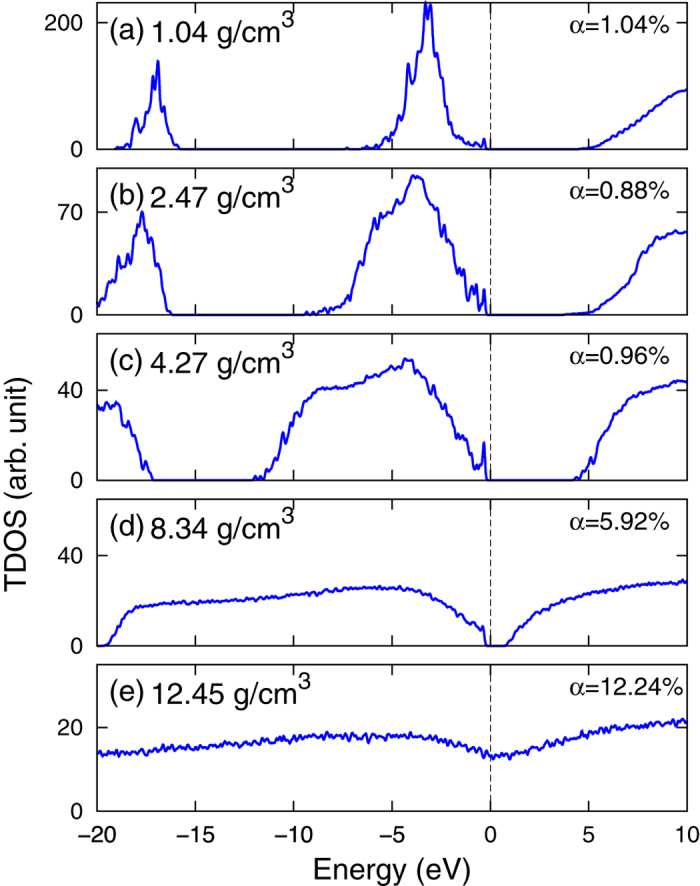
Total density of states(TDOS) of Ar plasma along the T = 10^4^ K isotherm, calculated using the FTDFT+MD method. The degree of ionization *α* is also displayed together with the TDOS.

Total density of electronic states (TDOS) of Ar plasma along the *T* = 10^4^ K isotherm is presented in Fig. 2. It is a convenient way to display the electronic structure and the IP. Note that the position of chemical potential in the figure has been shifted to the origin point of the energy scale. In Fig. 2(a–c), the TDOS have three separate parts associated with different atomic orbitals: The band below −15 eV comes from the 3*s* states, the segment in the middle is from the 3*p* states, and the continuum above the chemical potential is formed by the bottom of conduction bands. The 3*s* and 3*p* states in the figure are broadened significantly due to thermal effects and tunneling effects. The calculated IP is ~5 eV in a large range of density until *ρ* ~ 4.3 g/cm^3^, from which the IP starts to shrink to zero. This tendency is similar to that observed in KCl under shock compression^27^. Figure 2(d) displays the TDOS at *ρ* = 8.34 g/cm^3^, corresponding to an atomic distance comparable with the average diameter of the outer-most 3*p* orbital of Ar. The small IP (~0.8 eV) suggests that the Ar plasma is close to a nonmetal-metal transition point. The merging of the 3*s* and 3*p* states also implies a remarkable change in the electronic structure. Figure 2(e) shows the TDOS of a metallic state at *ρ* = 12.45 g/cm^3^. The disappearing of energy gap leads to an *α* over 12%, which is twice of that for *ρ* = 8.34 g/cm^3^ in Fig. 2(d). If one takes the nonmetal-metal transition as the indication of Mott effect, the variation of electronic structures displayed in Fig. 2 agrees with the usual understanding of Mott effect, i.e., the effect takes place where atomic distance is comparable with the diameter of the outer-most electronic orbital. No evidence is found to support Mott effect at lower density.

**Figure 3:**
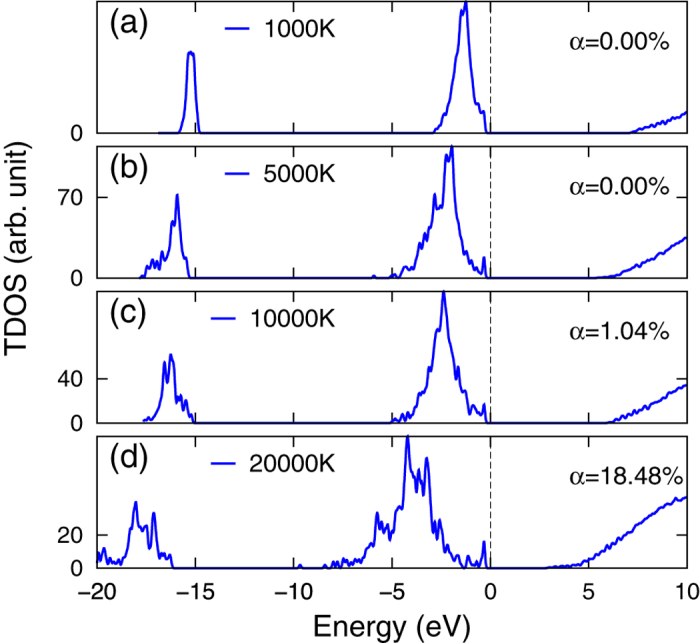
Total density of states (TDOS) of Ar plasma along the *ρ* = 1 g/cm^3^ isochore at selected temperatures, calculated using the FTDFT+MD method. *α* is also displayed to show the variation of ionization.

The influence of temperature is presented in Fig. 3, where TDOS of selected temperatures along the isochore of *ρ* = 1 g/cm^3^ are displayed. When temperature increases from *T* = 10^3^ K to *T* = 2 × 10^4^ K, the 3*s* and 3*p* TDOS are broadened by about twice, and the IP decreases smoothly from ~7 eV to ~4 eV. No abrupt change of IP is observed in the temperature range. As has been pointed out by the PPT model^2^ and the pressure ionization model^4^, Mott effect takes place below a critical temperature, at which thermal energy (effectively repulsive) and screened Coulomb energy (effectively attractive) arrive at a balance. So, when there is no indication of Mott effect at low temperature, the effect is less likely to be found at higher temperature. Even lower temperature gives rise to a negligible *α*, which is unlikely to reach an *α* ~ 1 as SBSL experiments suggested.

### Influence of Dynamical Screening

To understand the role of dynamical screening effect in determining electronic structures, which is not sufficiently accounted for in the FTDFT+MD method, we further calculate the ionization ratio of Ar under typical conditions with the influence of dynamical screening included using the TDDFT method. It gives an explicit description to the real-time evolution of excited electrons by solving the time-dependent Kohn-Sham equation using adiabatic local density approximation (ALDA), and has been applied to a few occasions, e.g., atomic collisions^45^, laser-solid interactions^33^, and optical responses of solids^46^, with reasonable results. Note that, because of the locality of ALDA^30^, long range excitation process is not fully described in the TDDFT+MD method. To take this process into consideration, a long range exchange correlation functional is necessary. In the TDDFT+MD method, only the temperature of ions are controlled by a Nosé-Hoover heat reservoir^47^, the electrons are thermalized through ion-electron interaction. It has been noticed that^48^, without external time-dependent sources, such as photons, it is extremely inefficient for the TDDFT+MD method to thermalize electrons from their ground states to a desired temperature through the ion-electron interaction^48^. A more rigorous discussion on the thermalization is provided by Modine and Hatcher^48^. Alternatively, as adopted in the work for Ar with a large band gap, one can boost the thermalization process by increasing the ion temperature artificially (10–20 times of the desired temperature in our work) at the early stage of the simulation. When the electron system arrives at a temperature close to the desired one, the ion temperature is switched back to the desired temperature. The electrons can then reach the desired state much faster. In the actual calculation, the optimal timing to switch the ion temperature is determined by a few of trials case by case. Details of the method are referred to the methods section.

**Figure 4:**
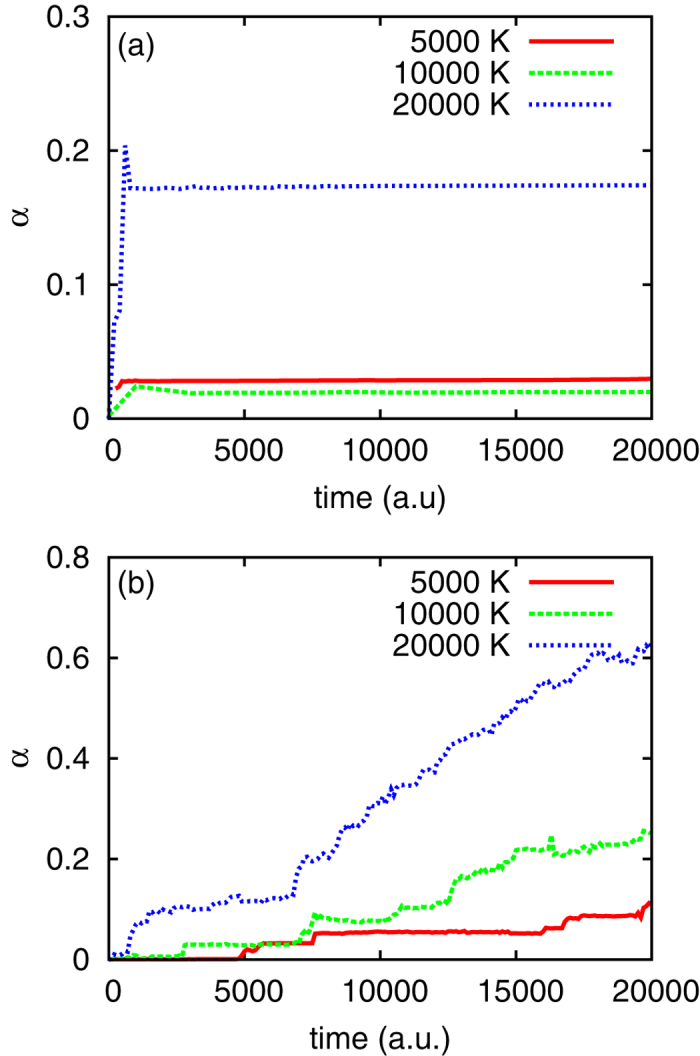
Degree of ionization *α* at *ρ* = 3.6 g/cm^3^ (**a**) and *ρ* = 10.5 g/cm^3^ (**b**), calculated using TDDFT+MD method. Degree of ionizations at various temperatures are distinguished by colors. They represent the evolution of ionization for typical non-metallic and metallic systems respectively. Note that different scales of degree of ionization are used in (**a**,**b**), and an artificial boost of ionization is employed at the early stage in the calculation of (**a**) to make the ionization reaches its equilibrium faster.

Figure 4 displays the evolution of *α* at two representative densities *ρ* = 3.6 g/cm^3^ and *ρ* = 10.5 g/cm^3^. Figure 4(a) is calculated with an artificial boost of ionization at the beginning to accelerate the calculation. It shows that the system has an *α* of 17.2% at T = 2 × 10^4^ K for *ρ* = 3.6 g/cm^3^, which is similar to the value calculated using the SCFVT method^39^ but much lower than the value (*α* ≥ 1) observed in the experiments. An indication of typical Mott effect is illustrated in Fig. 4(b) at *ρ* = 10.5 g/cm^3^. When the energy gap closes and the system becomes metallic, stepwise multi-ionization processes are observed, and *α* reaches 12% at 0.48 ps (20000 a.u.) for T = 5 × 10^3^ K without artificial boost to the ionization. Further increase in *α* is observed when temperature rises, and the ionization process does not stop in our longest calculation at T = 2 × 10^4^ K after 0.72 ps (30000 a.u.) In general, Fig. 4 shows that dynamical screening included in the TDDFT+MD calculation does not induce a sharp transition at low density, and therefore, the physical picture presented by the FTDFT+MD calculation is qualitatively reliable.

### Influences of Other Ion Species

**Figure 5:**
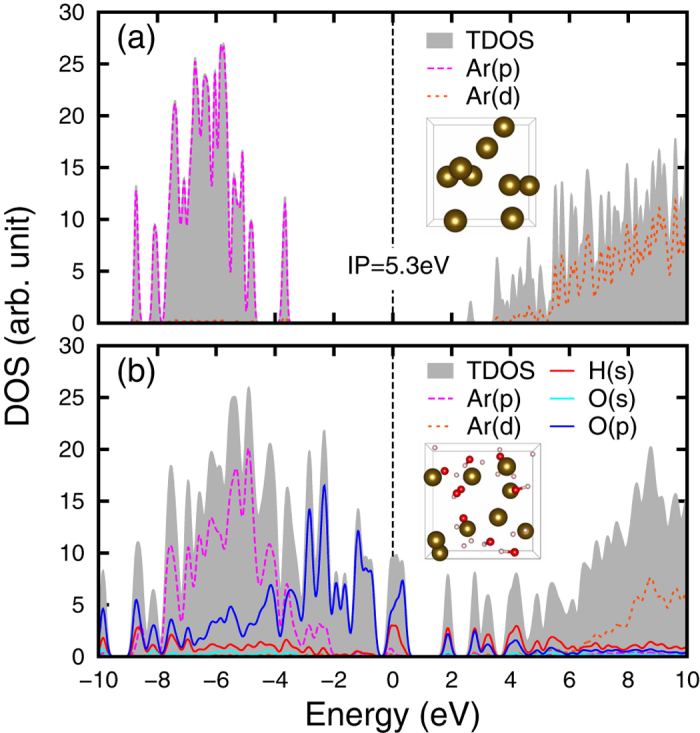
Total and projected density of states for pure Ar at *ρ* = 1.7 g/cm^3^ (**a**) and Ar mixed with H_2_O of the same number density (**b**). Both are calculated at T = 10^4^ K using the FTDFT+MD method. Ion configurations are also displayed in the insets, where golden spheres represent Ar, small white spheres are H, and red spheres are O.

From the perspective of electronic structures, we have shown that the strong ionization observed in the Ar SBSL experiments is quite unlikely the result of Mott effect of pure Ar plasma. However, it is still possible to obtain an effective IP small enough to meet the requirement of Mott effect when a mixture of plasmas composed of several ion species are considered. Energetically, this can be achieved by inserting energy levels of other ion species into the gap of Ar plasma. Indeed, as pointed out by chemical reaction models^49^ and experiments^20^, ionized water and other ion species from the solution can also exist inside the collapsing bubble in a SBSL experiment. Figure 5 shows the effect of water mixed with Ar plasma at *ρ* = 1.7 g/cm^3^, *T* = 10^4^ K. This state has a density close to the lowest density bound in our calculation at typical SBSL temperatures, and is expected to have the least possibility to present the Mott effect according to the trend illustrated in the FTDFT+MD and TDDFT+MD calculations. To distinguish the contribution of different ion species, the density of states is decomposed into projected density of states (PDOS) according to the composing elements (Ar, O, and H) and their electronic orbitals. For pure Ar plasma, as displayed in Fig. 5(a), a large IP of 5.3 eV is observed. When H_2_O at the same number density (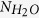 : *N*_*Ar*_ = 9 : 9) is introduced, as displayed in Fig. 5(b), the density of states near the chemical potential is then dominated by the *p* orbital of O, originated from OH^−^ radicals or H_2_O molecules. At this temperature (*T* = 10^4^ K), OH^−^ does not have enough energy to decompose into O^2−^ and H^+^. So, the contribution of O^2−^ is negligible. As a result, the energy gap in Ar plasma is filled by energy levels associated with ionized water, and the effective IP of the mixed Ar and H_2_O plasma is substantially reduced, making the strong ionization possible.

In summary, we show from the perspective of electronic structures that the strong ionization observed in the SBSL experiments is unlikely the result of Mott effect in a pure Ar plasma. Instead, a mixture of plasmas composed of Ar and water energetically favors the occurrence of strong ionization around T = 10^4^ K. Our first-principles investigation supports the traditional understanding of Mott effect, i.e., the Mott effect happens at the density where atomic distance is comparable with the diameter of the outer-most electronic orbitals. Although the detailed mechanism of Mott effect is still an open question, this investigation would help to have a better understanding of Mott effect as well as its relationship to SBSL experiments.

## Methods

FTDFT+MD simulations are carried out by the Vienna ab initio simulation package (VASP)^50^, where Perdew-Zunger parametrization of local density approximation (LDA) is used for the exchange and correlation potential^34^. The 3s and 3p electrons are explicitly treated as valence in the calculation. The population of electronic states is assigned according to Fermi-Dirac distribution, and electronic temperature is set the same as that of ions to ensure local thermodynamical equilibrium^29^. Electron-ion interactions are modeled using the projector augmented wave (PAW) pseudo-potentials^51^. Wave functions are expanded up to a cutoff energy of 400 eV. Through the FTDFT+MD simulations, periodic boundary conditions are enforced and the Brillouin zone is sampled by the Γ point. Note that in the calculation of density of states, a 4 × 4 × 4 Monkhorst-Pack k-point grid is used to ensure the accuracy. For pure Ar plasma, 64 atoms are included in the FTDFT+MD simulation, while for the mixture of H_2_O and Ar, a total number of 36 atoms are used. The time step varies from 0.4–1 fs depending on the density and temperature. Each simulation lasts for 8000–20000 time steps, and the results presented are averaged over the last 2000 steps with a time interval of 200 steps. Degree of ionization is calculated following ref. 27, and the IP is calculated as the energy difference between the two adjacent Kohn-Sham orbitals across the chemical potential at finite temperature, or as the energy difference between the lowest unoccupied and the highest occupied Kohn-Sham orbital at *T* = 0. Note that the Kohn-Sham energy level is interpreted as an approximation to the quasiparticle energy following the convention of electronic structure calculation for extended systems^34^, in order to keep in line with the many-body quantum-statistical theory of plasmas^2^.

TDDFT+MD calculations are carried out using the OCTOPUS code^31^. ALDA functional is used to describe the exchange-correlation interaction. A total number of 8 Ar atoms are treated in the calculation through norm-conserving Troullier-Martins pseudopotential^52^, with each having eight valence electrons. Periodic boundary conditions are used throughout, and wave functions are represented using a three-dimensional spatial grid with 20 points on each direction (approximately equivalent to an energy cutoff of 50 Ry). The Brillouin zone is sampled by a 2 × 2 × 2 Monkhorst-Pack shifted grid. In the calculation, ionic temperature is controlled through the Nosé-Hoover heat reservoir^47^, while wave functions of electrons propagate according to the time-dependent Kohn-Sham equation with a time step about 0.1 a.u. (1 a.u. = 0.024 fs) up to 30000 a.u. To ensure the stability of this large time step, the propagator is calculated using the a Lanczos method^53^ up to an order of 30, and the error of the propagator is kept less than 10^−8^ in each step.

In the framework of TDDFT, the degree of ionization *α*(*t*) is calculated as the charge excited from the ground state per atom. By projecting the time-dependent wave functions onto the ground-state Kohn-Sham wave functions at that instant, *α*(*t*) can be evaluated as


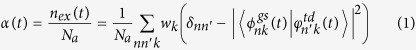


where *N*_*a*_ is the number of atoms, *n*_*ex*_ is the average number of excited electrons, *w*_*k*_ is the weight of each k-point in the summation of Brillouin zone, 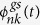 is the ground state Kohn-Sham wave functions calculated using the ion configuration at instant *t*, and 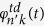 is the time-dependent electronic wave functions.

## Additional Information

**How to cite this article**: Kang, W. *et al.* First-Principles Investigation to Ionization of Argon Under Conditions Close to Typical Sonoluminescence Experiments. *Sci. Rep.* **6**, 20623; doi: 10.1038/srep20623 (2016).
